# CATH: an expanded resource to predict protein function through structure and sequence

**DOI:** 10.1093/nar/gkw1098

**Published:** 2016-11-29

**Authors:** Natalie L. Dawson, Tony E. Lewis, Sayoni Das, Jonathan G. Lees, David Lee, Paul Ashford, Christine A. Orengo, Ian Sillitoe

**Affiliations:** Institute of Structural and Molecular Biology, University College London, Gower Street, London, WC1E 6BT, UK

## Abstract

The latest version of the CATH-Gene3D protein structure classification database has recently been released (version 4.1, http://www.cathdb.info). The resource comprises over 300 000 domain structures and over 53 million protein domains classified into 2737 homologous superfamilies, doubling the number of predicted protein domains in the previous version. The daily-updated CATH-B, which contains our very latest domain assignment data, provides putative classifications for over 100 000 additional protein domains. This article describes developments to the CATH-Gene3D resource over the last two years since the publication in 2015, including: significant increases to our structural and sequence coverage; expansion of the functional families in CATH; building a support vector machine (SVM) to automatically assign domains to superfamilies; improved search facilities to return alignments of query sequences against multiple sequence alignments; the redesign of the web pages and download site.

## INTRODUCTION

CATH-Gene3D, established in the mid 1990s, is a publicly-accessible, online resource providing a protein domain structure classification (1) (http://www.cathdb.info). CATH identifies protein domain structures within protein three-dimensional (3D) structures from the Protein Data Bank (PDB) (2) and assigns domains sharing evolutionary similarities to the same superfamily within the CATH hierarchical structure classification. The structural hierarchy allows these superfamilies to be organised within structural space, the letters CATH represent the levels in that hierarchy: Class, Architecture, Topology and Homologous superfamily.

Additional protein domain sequences with no known structure are identified from UniProtKB (3) and Ensembl (4) protein sequences and classified within our sister resource Gene3D (5). Hidden Markov model (HMM) technology (6) is then employed to create profiles, or evolutionary fingerprints, seeded from these CATH structural domains. All known protein sequences are then scanned against these HMMs and where significant matches are found, domain boundaries and evolutionary relationships are inferred onto the query protein sequence.

CATH-Gene3D has recently become an ELIXIR-endorsed resource. The ELIXIR programme has a number of objectives in developing bioinformatics resources, including: training, developing data standards, collaborations, developing tools and supporting innovation (https://www.elixir-europe.org/). The CATH-Gene3D resource is contributing to a current ELIXIR research programme, EXCELERATE, in which the CATH-Gene3D HMMs are being used to assign domain structure and function annotations for metagenome sequences in the marine metagenome data use case.

## CATH v4.1 RELEASE HIGHLIGHTS

Since the last release of CATH (v4.0), an additional ∼73 000 domains of known structure and over 4000 sequence families (i.e. families of homologous domains with at least 35% sequence identity) have been incorporated.

## CATH-B

Creating official releases of CATH (e.g. v4.1) is an important part of our resource pipeline: it provides a static target to which other resources (both internal and external) can consistently reference their own data. Once the domain definitions and classifications for a particular CATH release have been ‘frozen’, the release protocol goes on to generate additional calculations, annotations and analyses. The extra data provided from these steps are integral both for internal research projects in the future and the backwards compatibility required by the external scientific community. However, this release process also increases the time between the very latest structures being released in the PDB and the time annotations are made available in the latest release of CATH-Gene3D.

To address this, we also provide access to the very latest putative CATH annotations in CATH-B. This list of domain definitions and superfamily assignments is updated daily (1) and is available to download in a number of different formats. Over 414 000 domains are present in CATH-B, adding an additional ∼100 000 domains to the v4.1 release of CATH-Gene3D. There has also been a significant increase in the number of homologous superfamilies putatively classified in CATH as a result of the new support vector machine (SVM) method (see ‘New Data in CATH’ Section).

Since a number of additional checks and analyses are performed when a release is frozen, it is possible that some assignments may change from CATH-B (e.g. additional evidence may allow two superfamilies to be merged). However, in practice, only 0.15% of all domain boundaries and 0.02% of all superfamily assignments were seen to change during the last release process (from CATH-B to v4.1). As such, these ‘putative’ assignments in CATH-B should be considered safe to use.

### CATH FunFams

FunFams or Functional Families are sub-clusters of domains (structures and sequences) within homologous superfamilies that are likely to have highly similar structures and functions. FunFams are generated by agglomerative hierarchical clustering of domain relatives within a superfamily using the Genome Modelling and Model Annotation (GeMMA) algorithm (7) followed by optimal partitioning of the resulting superfamily clustering tree by the automated family classification method, FunFHMMer (8). FunFHMMer uses evolutionary patterns within cluster sequence alignments such as highly conserved positions and specificity-determining positions to distinguish between clusters that are likely to share the same function and vice-versa, thereby determining an optimal cut of the superfamily tree.

Sub-classification of the CATH superfamilies into FunFams helps to capture the functional diversity within a superfamily and allows comparison of functional sites between relatives. This provides valuable insights into evolutionary mechanisms underlying changes in function (9,10). It also helps to provide function annotations of greater precision for more uncharacterised proteins than other domain-based resources (8). Functional annotations predicted using CATH FunFam assignments were ranked among the top five function prediction methods (out of 126 methods) in the Critical Assessment of Function Annotation experiment 2 (11) for both ‘molecular function’ and ‘biological process’ prediction.

To update the FunFams, the CATH superfamilies were first populated with updated sequence domains from our sister resource Gene3D (v14). These domain sequences were obtained by scanning UniProtKB and Ensembl sequence data against the CATH v4.1 HMMs (constructed for each S35 cluster representative in a CATH superfamily using the HMMER3 software suite (6)). Domains assigned to a given CATH superfamily in v4.1 were mapped to equivalent domains assigned to FunFams in v4.0. Since it is possible that domain boundaries change between versions of CATH (due to updated software, updated HMMs, additional sequence data, etc.), any domain that was seen to change significantly (i.e. overlap < 70%) was removed from the existing FunFam.

These mapping issues affected ∼17% of the Gene3D sequences assigned in v4.1. Many of the domain sequences that changed significantly came from singleton FunFams (i.e. FunFams with a single member) or FunFams with low populations, which resulted in removing ∼17 000 FunFams. As a result, CATH-Gene3D v4.1 now comprises 92 882 FunFams from 2737 superfamilies. Approximately 12% of the FunFams in v4.1 have increased in population by 10% or more since v4.0.

After refreshing FunFams with the updated domain sequences, new FunFam HMMs were used to search against the pool of novel domain sequences. Inclusion thresholds were determined for each FunFam as described in (8) and new domains were added to existing FunFams if the match surpassed the inclusion threshold.

CATH currently classifies over 21 million domain sequences into FunFams within superfamilies. Since functional terms have been integrated from Gene3D (i.e. Gene Ontology (GO) (12) and EC classification (13) terms), this constitutes 27 459 unique functional terms associated with FunFam sequences (4504 different EC terms and 22 955 different GO terms). Functional information is displayed on dedicated web pages for each FunFam. CATH-FunFams considerably increase the amount of functional information available for sequences in important model organisms. For example, the current FunFams provide functional annotations for ∼50% of human proteins compared to 8.7% of the human proteins that have been experimentally characterised to date (UniProt-GOA, September 2016, https://www.ebi.ac.uk/GOA).

For each FunFam, a multiple sequence alignment (MSA) is constructed using MAFFT (14) and this is displayed on the web page (see ‘Improvements to Web Pages’ Section). An entropy-based method, Scorecons (15) is used to determine the information content of each MSA according to the diversity of positions (DOPs) score, which also provides the degree of conservation of each position in the alignment. Each column in an MSA has a conservation score calculated between 0 (i.e. completely unconserved) and 1 (i.e. completely conserved). The DOPs score captures the amount of diversity in an MSA by considering all of the different conservation scores and their frequencies, and provides a value between 0 (i.e. zero diversity) and 100 (i.e. no alignment positions have the same conservation score). An MSA with a score of at least 70 is considered suitable diverse for analysis.

#### Frozen FunFams

Highly informative FunFams (DOPs score ≥ 70) have sufficient sequence diversity to identify positions highly conserved throughout evolution. There are currently 14 930 (16%) highly informative FunFams from 2053 (75%) superfamilies, comprising 18 110 832 (∼85%) sequences. These FunFams have been frozen and will be maintained through future updates. The remaining FunFams will be regularly updated, i.e. sequences from all non-frozen FunFams will be combined with any new sequences added by future Gene3D scans. They will subsequently be re-clustered using our GeMMA protocol (7) and new functional families identified using the FunFHMMer algorithm (8).

#### Displaying relationships between FunFams in a superfamily

**Figure 1.**
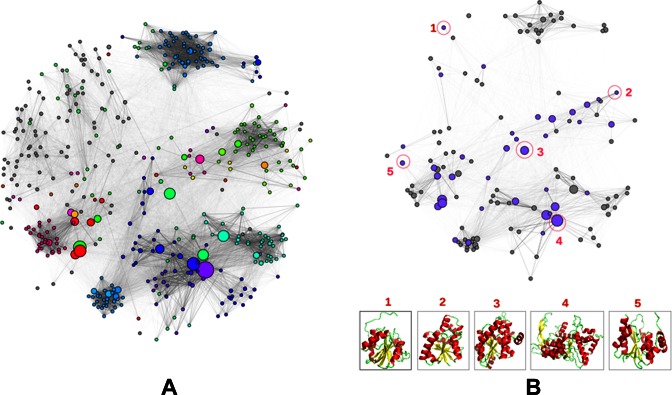
Visualisation of functional and structural diversity in the HUP superfamily using Cytoscape (25). The nodes in the network represent FunFams and the edges represent sequence similarities between the FunFam HMMs calculated using Profile Comparer (PRC) (16). The size of the nodes (FunFams) reflects their number of sequences and the nodes are linked by edges if the similarity of their HMMs is above a PRC score of 10. (**A**) This network highlights the functional diversity of the HUP superfamily where all nodes are coloured according to the EC numbers of their constituent sequences and grey nodes indicate those without any EC annotation (including non-enzymes). (**B**) This network shows the available structure data among the FunFams with high information content in the HUP superfamily. The purple coloured nodes indicate FunFams with known structure and the grey nodes indicate FunFams without any known structure. Structural representatives of selected FunFams (encircled and numbered in red) are shown at the bottom of the figure to highlight the structural diversity of the superfamily.

Many highly-populated superfamilies in CATH incorporate considerable structural and functional diversity. A comprehensive summary of sequence, structure and functional diversity in a CATH superfamily can be provided by the use of FunFam similarity networks. For all CATH domain superfamilies having two or more FunFams, superfamily networks have been constructed in which FunFams are represented by nodes and the edge distances correspond to the sequence similarity between the FunFam HMMs assessed using profile comparer (PRC) (16). For example, Figure 1 shows two networks for the structurally and functionally diverse HUP superfamily that are useful for understanding how function has been modulated by sequence or structure changes between the FunFams.

#### FunFam naming method

FunFam names are derived from an analysis of the UniProtKB descriptions assigned to each of the member sequences. The intention was to provide an algorithm that selects a single UniProtKB description that best represents all the sequences in the cluster. Ideally this would provide FunFams with names that are unique, specific and biologically relevant. Since some FunFams can contain a large number of sequences with very different UniProtKB descriptions, it was also important that the given name tries to take into account diversity as well as specificity.

For each FunFam cluster:

- Remove redundancy from very similar sequences (CD-HIT (17) at 95% sequence identity)
- Normalise all the terms (words) seen in all the UniProtKB descriptions: removing trivial terms and standardising nomenclature where possible
- Provide each term with a score based on the frequency across the entire cluster
- Score each UniProtKB description by summing the values of each of the constituent terms
- Normalise the scores to favour descriptions where the number of terms is closest to the average number of terms seen in the cluster (avoiding over-specific names)

## NEW DATA IN CATH

### Homology assignments using an SVM-based method

In order to keep pace with the number of incoming PDB structures, it is important to be able to use automatic methods to inherit annotations from previous curations (e.g. domain boundaries and homology assignments). However, incorporating automatic annotations without appropriate supervision can introduce inaccuracies, and these inaccuracies can be compounded when automated annotations are chained together. To address this, a number of internal checks are made on putative domain boundary assignments. If any of these checks fail, the chopping is sent to be manually validated; a process that often results in the suggested boundary being adjusting by one or two residues. While this curation process can be time-consuming, it has been integral in allowing us to build accurate, high-quality HMMs for each sequence family (S35), seeded by the sequence of a robustly assigned representative domain structure. Currently domains in ∼5% of new depositions fail these checks and have to be manually ‘chopped’.

Assigning domains to superfamilies in CATH can also be difficult if the query domain in question is a remote homologue to classified relatives. High quality manual assignments can be used in such cases, but they are time intensive and require expertise. To help overcome these issues, a supervised SVM has recently been implemented. This combines all available data on sequence and structural similarity for known relatives in CATH-Gene3D to produce a powerful predictor that can identify remote evolutionary relationships. Similarity measures are collected using a variety of metrics from two algorithms: SSAP (18) and PRC (16).

The SVM was benchmarked using a 50/50 set of homologous and non-homologous domain pairs. A total of 21 576 pairs of CATH domains were evenly divided into 10 788 homologous pairs and 10 788 non-homologous pairs. This data set was constructed similar to the procedure in (19), where ‘easy’ positives are removed to produce a list containing no two domains with high sequence identity between them. Also, no two domains had a SSAP score above 80. This provided a list of 3788 domains, from which the pairs were generated. The 10 788 negatives (i.e. non-homologues) were selected from across the CATH classification as follows:

- 5754 (∼8/15ths) are T-level matches
- 2877 (∼4/15ths) are A-level matches
- 1438 (∼2/15ths) are C-level matches
- 719 (∼1/15th) are domains in different classes.

The negative pairs were randomly selected from across this set. The Class (C)-level of CATH represents the types of structural content present in a domain, e.g. mainly alpha helical, mainly beta sheet, alpha-beta. The A-level represents similarities in domain architectures, i.e. the 3D arrangements of secondary structure elements. The T-level represents similarities in the topology/fold, i.e. the 3D arrangement and the connections between secondary structure elements. Finally, the H-level represents the evolutionary related members grouped together in homologous superfamilies.

**Figure 2.**
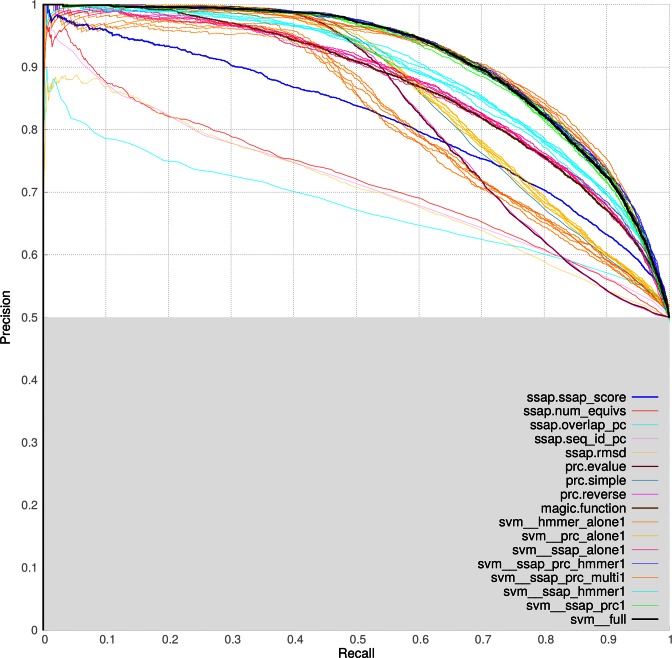
The precision and recall results from the first benchmark where equal sets of carefully chosen homologous and non-homologous domain pairs were using for the training and testing of various scoring algorithms.

The graph in Figure 2 represents the results of testing multiple scoring methods. Groups of lines in the same colour represent repeated SVM experiments on a random 50/50 split of data into training and testing. The green lines represent the PRC and SSAP score SVMs, which was chosen as the final SVM. Adding other algorithms does not make enough difference to the results to justify adding in extra complexity. The black lines represent the final SVM results.

Further work was done in comparing/combining structural comparison scores in a SVM. These results combined with those above indicated that additional methods (e.g. Structal (20), TM-Align (21)) did not significantly improve performance.

When assigning domains to a CATH superfamily using the SVM algorithm, the following protocol was used. Firstly, find the best SVM hit to an assigned domain with an SVM score that reaches the optimal benchmarked threshold (3.47) and a SSAP overlap of at least 70%. If there is such a hit, and its match does not belong to a node known to be problematic (e.g. the beta propellers that often provide false positives), assign the domain to the hit's superfamily. Otherwise, find the SSAP hit with the highest SSAP score, where the SSAP score ≥ 70 and the SSAP overlap ≥ 60%. If this hit exists, assign the domain to a brand new superfamily within the match's fold (i.e. create a new superfamily in the fold and assign to that). Otherwise, document the domain and its match information for manual assessment as it may belong to a new fold.

The SVM has already been deployed to tackle as much as possible of the backlog of unassigned domains. This caused a bump in the growth of CATH; in ∼3 months that the assignments were performed on this backlog (December 2015–March 2016), the number of assigned domains increased by 67 000 (∼20%).

### Improvements to the web pages

#### The home page

**Figure 3.**
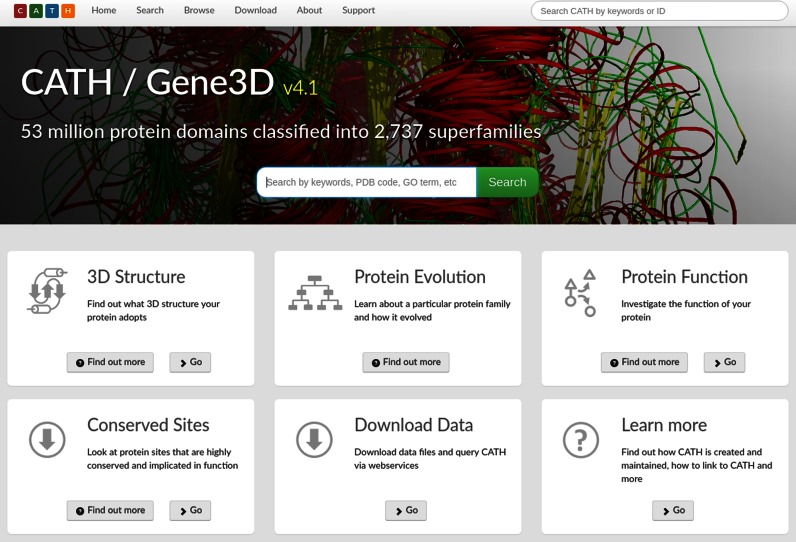
Redesigned home page for CATH-Gene3D.

Introductory information is provided on: 3D structure; protein evolution; protein function; and conserved sites by selecting the new ‘Find out more’ buttons (Figure 3). These pop-up windows also explain how to use CATH-Gene3D to find the information that the user may be looking for. Also provided on the home page are the latest statistics for the most recent version of CATH and for CATH-B, together with download links.

#### Sequence search

Users can search for matches to structural domains and functional families. The user's query sequence is scanned against all sequences in CATH using BLAST (22) to find the most closely related match.

#### Sequence alignment/structure viewer

**Figure 4.**
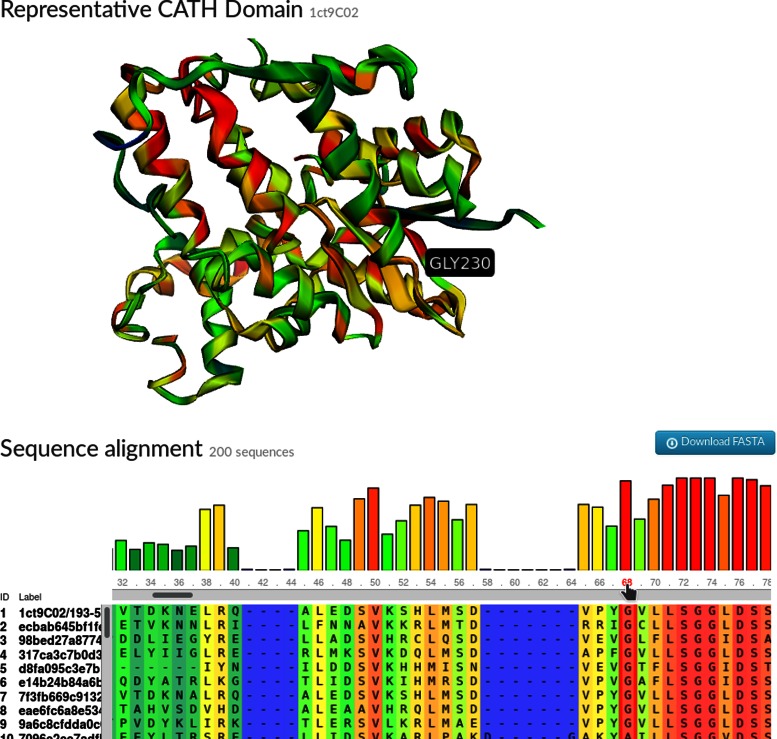
Section of the CATH web pages displaying the multiple sequence alignment for a CATH FunFam (3.40.50.620/FF/89168) underneath the structural domain chosen to represent the cluster (1ct9C02). The degree of sequence conservation is highlighted on a sliding colour scale on both the alignment and the structure (blue-red signifying low-high conservation). Clicking on the alignment positions on the alignment highlights accompanying residues in the structure. The sequence alignment and 3D structure are displayed using open source tools: MSAViewer and 3DMol.js, respectively.

A new sequence alignment to structure viewer has been introduced to the FunFam pages to more clearly highlight how conservation patterns in the sequence alignment map to the 3D structure (Figure 4). This feature combines MSAViewer (23), a multiple sequence alignment viewer and 3DMol.js, a 3D molecular structure viewer (24).

### Open source tools on GitHub

CATH-Gene3D software tools for protein structure analysis have been made available under the GNU GPL v3 License at GitHub (https://github.com/UCLOrengoGroup/cath-tools). For example, the latest version of SSAP, the CATH structure comparison algorithm can be downloaded as cath-ssap. Other tools include cath-superpose, which superposes multiple protein structures, and cath-resolve-hits, a tool that takes a list of hits to a protein and quickly identifies the optimal subset that constitute a single, consistent architecture.

Whenever any changes are pushed to the GitHub repository, the new version's documentation is built by Read the Docs (http://cath-tools.readthedocs.io/en/latest/) and its code is built by Travis-CI (https://travis-ci.org/UCLOrengoGroup/cath-tools). The Travis build includes build-verification tests containing over 250 000 test-assertions in over 700 test-cases. If the build and tests are successful, the resulting Ubuntu executable files are made available for public download. At present, binaries built on Ubuntu 12.04 Linux are provided; the code is developed under both GCC and Clang with strict compilation settings (including failing on warnings). We welcome engagement from users interested in using the code on other mainstream platforms.

### Outreach with social media

To inform users of our ongoing work in developing and updating CATH through CATH-B, we provide weekly tweets on newly identified domains and newly classified domains (@CATHDatabase). These automated tweets also provide a link to an example domain that has been classified within the last week.

## CONCLUSION

This article describes the latest release of CATH-Gene3D v4.1 that contains over 300 000 structural domains and over 53 million predicted domain sequences. This represents a significant increase in structural and sequence coverage.

We have expanded our functional families (FunFams) considerably to include new structural and sequence domains. We have also created a subset of FunFams deemed to be of sufficiently high quality to be ‘frozen’. The identifiers for these ‘frozen’ FunFam clusters will remain consistent between versions of CATH, allowing external resources to retain annotations and links.

While the process of identifying fold matches is relatively easy, defining homology can be more difficult. To aid in the process of assigning domains to a homologous superfamily, an SVM has been benchmarked and implemented. This has greatly increased the amount of putative domain assignments and superfamily numbers available through the daily release of CATH-B.

A large amount of work has been done to improve the clarity and accessibility of the web pages. The home page has been redesigned to provide users instant access to information on how to retrieve the information they require from CATH-Gene3D. Search facilities have been improved to allow users to align their query sequences against closest structural homologues and functionally related alignments. A new viewer has been added to the website for viewing MSA data and 3D structural domain data. The download site has also been redesigned to provide more intuitive access to data.

We encourage users to contact us with suggestions for new features by raising issues on GitHub: https://github.com/UCLOrengoGroup/cath-todo.

## References

[1] Sillitoe I., Lewis T.E., Cuff A., Das S., Ashford P., Dawson N.L., Furnham N., Laskowski R.A., Lee D., Lees J.G. (2015). CATH: comprehensive structural and functional annotations for genome sequences. Nucleic Acids Res..

[2] Rose P.W., Prlić A., Bi C., Bluhm W.F., Christie C.H., Dutta S., Green R.K., Goodsell D.S., Westbrook J.D., Woo J. (2015). The RCSB Protein Data Bank: views of structural biology for basic and applied research and education. Nucleic Acids Res..

[3] The UniProt Consortium (2014). UniProt: a hub for protein information. Nucleic Acids Res..

[4] Aken B.L., Ayling S., Barrell D., Clarke L., Curwen V., Fairley S., Fernandez Banet J., Billis K., García Girón C., Hourlier T. (2016). The Ensembl gene annotation system. Database (Oxford)..

[5] Lam S.D., Dawson N.L., Das S., Sillitoe I., Ashford P., Lee D., Lehtinen S., Orengo C.A., Lees J.G. (2016). Gene3D: expanding the utility of domain assignments. Nucleic Acids Res..

[6] Finn R.D., Clements J., Arndt W., Miller B.L., Wheeler T.J., Schreiber F., Bateman A., Eddy S.R. (2015). HMMER web server: 2015 update. Nucleic Acids Res..

[7] Lee D.A., Rentzsch R., Orengo C. (2010). GeMMA: functional subfamily classification within superfamilies of predicted protein structural domains. Nucleic Acids Res..

[8] Das S., Lee D., Sillitoe I., Dawson N.L., Lees J.G., Orengo C.A. (2015). Functional classification of CATH superfamilies: a domain-based approach for protein function annotation. Bioinformatics.

[9] Furnham N., Dawson N.L., Rahman S.A., Thornton J.M., Orengo C.A. (2016). Large-scale analysis exploring evolution of catalytic machineries and mechanisms in enzyme superfamilies. J. Mol. Biol..

[10] Lee D., Das S., Dawson N.L., Dobrijevic D., Ward J., Orengo C. (2016). Novel computational protocols for functionally classifying and characterising serine beta-lactamases. PLoS Comput. Biol..

[11] Jiang Y., Oron T.R., Clark W.T., Bankapur A.R., D'Andrea D., Lepore R., Funk C.S., Kahanda I., Verspoor K.M., Ben-Hur A. (2016). An expanded evaluation of protein function prediction methods shows an improvement in accuracy. Genome Biol..

[12] Ashburner M., Ball C.A.A., Blake J.A.A., Botstein D., Butler H., Cherry J.M.M., Davis A.P.P., Dolinski K., Dwight S.S.S., Eppig J.T.T. (2000). Gene Ontology: tool for the unification of biology. Nat. Genet..

[13] Webb E. (1992). Enzyme nomenclature 1992.

[14] Katoh K., Standley D.M. (2013). MAFFT multiple sequence alignment software version 7: improvements in performance and usability. Mol. Biol. Evol..

[15] Valdar W.S.J. (2002). Scoring residue conservation. Proteins.

[16] Madera M. (2008). Profile comparer: a program for scoring and aligning profile hidden markov models. Bioinformatics.

[17] Fu L., Niu B., Zhu Z., Wu S., Li W. (2012). CD-HIT: accelerated for clustering the next-generation sequencing data. Bioinformatics.

[18] Orengo C.A., Taylor W.R. (1996). SSAP: sequential structure alignment program for protein structure comparison. Methods Enzymol..

[19] Koussounadis A., Redfern O.C., Jones D.T., Orengo C., Michie A., Jones S., Jones D., Swindells M., Thornton J., Murzin A. (2009). Improving classification in protein structure databases using text mining. BMC Bioinformatics.

[20] Kolodny R., Koehl P., Levitt M. (2005). Comprehensive evaluation of protein structure alignment methods: scoring by geometric measures. J. Mol. Biol..

[21] Zhang Y., Skolnick J. (2005). TM-align: a protein structure alignment algorithm based on the TM-score. Nucleic Acids Res..

[22] Camacho C., Coulouris G., Avagyan V., Ma N., Papadopoulos J., Bealer K., Madden T.L. (2009). BLAST+: architecture and applications. BMC Bioinformatics.

[23] Yachdav G., Wilzbach S., Rauscher B., Sheridan R., Sillitoe I., Procter J., Lewis S.E., Rost B., Goldberg T. (2016). MSAViewer: interactive JavaScript visualization of multiple sequence alignments. Bioinformatics.

[24] Rego N., Koes D. (2015). 3Dmol.js: molecular visualization with WebGL. Bioinformatics.

[25] Kohl M., Wiese S., Warscheid B. (2011). Cytoscape: software for visualization and analysis of biological networks. Data Mining in Proteomics: From Standardsto Applications.

